# Application of biomimetic double-layer biofilm stent in arthroscopic rotator cuff repair: A protocol of randomized controlled trial

**DOI:** 10.1097/MD.0000000000023960

**Published:** 2021-01-08

**Authors:** Liang Ma, Yongtao Xu, Xiaolong Xu, Qin Pan, Yongtao Xu

**Affiliations:** Jingzhou Central Hospital, The Second Clinical Medical College of Yangtze University, Jingzhou, Hubei province, China.

**Keywords:** arthroscopic rotator cuff repair, bionic double-layer biofilm scaffold, protocol, randomized controlled trial, rotator cuff tear injury, tendon-bone healing

## Abstract

**Background:**

Rotator cuff injury is the most common cause of shoulder dysfunction. Despite the continuous advancement of surgical techniques, the incidence of re-tearing after rotator cuff repair is still high. The main reason is that it is difficult to reconstruct the normal tendon bone interface and the process is slow, and the application of tissue engineering technology can promote tendon and bone healing. This study will evaluate the effect of the bionic double membrane stent on the rotator cuff healing after arthroscopic rotator cuff repair.

**Methods:**

This is a prospective randomized controlled trial to study the effect of biomimetic double-layer biofilm stent on rotator cuff healing. Approved by the clinical research ethics committee of our hospital. The patients were randomly divided into 1 of 2 treatment options: (A) a biomimetic double-layer biofilm stent group and (B) a non-bionic dual-layer biofilm stent group. Observation indicators include: visual analog scale score, University of California Los Angeles score, American Shoulder & Elbow Surgeons score and Constant-Murley score. Data were analyzed using the statistical software package SPSS version 16.0 (Chicago, IL).

**Discussion:**

This study will evaluate and evaluate the effect of the bionic double-layer membrane stent on the rotator cuff healing after arthroscopic rotator cuff repair. The results of this experiment will provide new treatment ideas for promoting rotator cuff tendon bone healing.

**OSF Registration number:**

DOI 10.17605/OSF.IO/FWKD6

## 1 Introduction

Rotator cuff tear refers to the continuous interruption of cuff like structure formed by the tendons of supraspinatus, infraspinatus, subscapularis and teres minor, due to trauma, degeneration and strain, impact, blood supply damage and other reasons, which causes shoulder pain and limited movement as the main clinical manifestations. It mostly occurs in middle-aged and elderly people, with an incidence rate of 6% in the younger than 60-year-old age group, and 30% in the older than 60-year-old age group,^[1]^ In asymptomatic people, the incidence also increases with age.^[2]^ The treatment of rotator cuff injury is complicated, and 1-stage repair is feasible for patients who have failed non-surgical treatment.^[3]^ Arthroscopic rotator cuff repair has become the “gold standard” for rotator cuff injury repair, and has the advantages of less trauma and fewer complications.^[4]^

With the deepening of the understanding of the disease and the development of surgical techniques, the rotator cuff repair has achieved satisfactory clinical results, but the postoperative imaging follow-up found that the success rate of biological repair of anatomical structures is low.^[5]^ There are many reasons for the failure. The main reason is that it is difficult to reconstruct the normal tendon-bone interface and the process is slow. Compared with the normal fibrocartilage bone-tendon junction, the scar connection formed after traditional surgery has significantly lower strength,^[6]^ Although this kind of scar can reshape the fibrocartilage structure, the process often takes as long as 6 months to 1 year.^[7]^ In order to make postoperative tendon healing more firm and rapid, it is necessary to find and apply new methods to provide an ideal condition to improve the activity of tissue cells around the rotator cuff healing site and rebuild the biomechanical function of the tissue.

At present, biological methods and materials based on growth factors, repair scaffolds, and stem cells have become the research hotspots of strategies to promote rotator cuff tendon bone healing.^[8–10]^ The biomimetic double-layer biofilm scaffold is a biomimetic periosteum that combines growth factors and bioactive stem cells. It controls the release of bone morphogenetic protein 2 (BMP-2) and vascular endothelial growth factor (VEGF) and platelet derived growth factor (PDGF) form specific expression, combined with the differentiation of bone marrow mesenchymal stem cells to help the regeneration of cartilage at the tendon-bone interface and promote tendon-bone healing.

In this study, a randomized controlled trial will be used to observe the effect of the biomimetic double-layer biofilm stent on the healing of the rotator cuff after arthroscopic rotator cuff repair, and to evaluate its long-term efficacy through follow-up and explore its effectiveness in promoting tendon bone healing after rotator cuff repair.

## 2 Materials and methods

### 2.1 Study design

**Figure 1:**
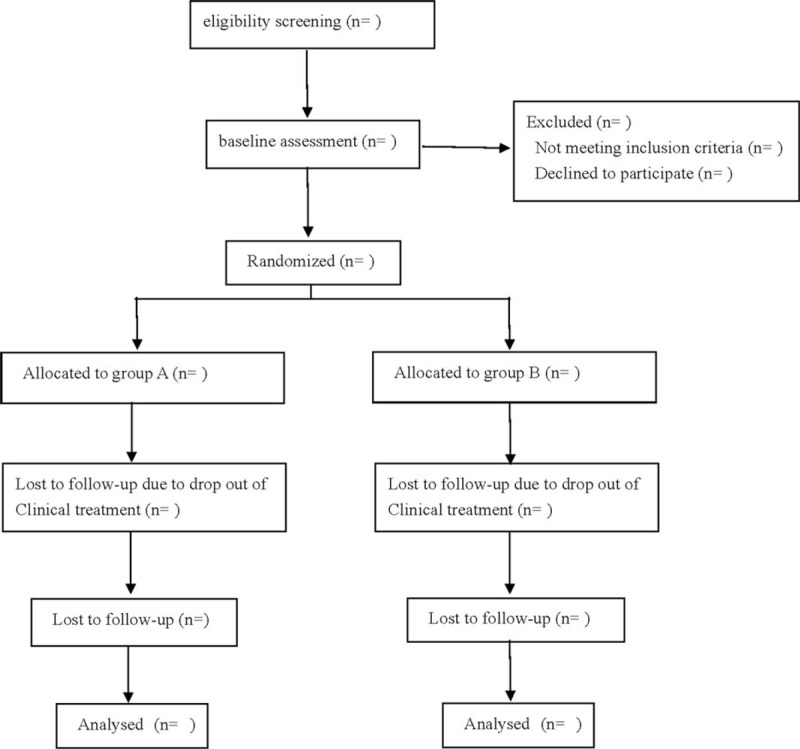
Flow diagram.

This is a prospective single-center randomized controlled trial to study the effect of a biomimetic double-layer biofilm stent on the healing of the rotator cuff after arthroscopic rotator cuff repair. The research protocol will follow the Declaration of Helsinki and comprehensive trial reporting standards.^[11]^ The flow chart is shown in Figure 1. And the study has been approved by the clinical research ethics committee of our hospital.

### 2.2 Ethics and registration

This research program is in accordance with the Helsinki Declaration and approved by the Clinical Research Ethics Committee of our hospital. This protocol has been registered in open Science Framework (OSF) (Registration number: DOI 10.17605/OSF.IO/FWKD6). All patients need to sign a written informed consent before they are randomly assigned to continue the trial.

### 2.3 Sample size

The calculation of the sample size is based on the visual analog scale (VAS)score results 1 month after the operation. According to the pre-test results, the average score of the experimental group is estimated to be 3.4 points and the standard deviation is 1.8. The control group averaged 4.6 and the standard deviation is 1.9. This study is a non-inverse design, 1-sided test, α =0.025, power = 90%, through calculation, each group needs 50 patients in total, and the estimated withdrawal rate is 20%. Each group will include 63 patients.

### 2.4 Patients

Inclusion criteria: ①Age 45 to 70 years; ②Shoulder joint pain, active flexion and lifting weakness, affecting work and daily life, non-surgical treatment for more than 6 months, and non-surgical treatment for more than 6 months; MRI examination confirmed full-thickness rotator cuff tear ③Patients undergoing arthroscopic rotator cuff repair for the first time.

Exclusion criteria: ①Partial tear; ②Huge or irreparable tear; ③With nerve injury; ④Biceps long head tendon injury; ⑤Shoulder joint osteoarthritis, rheumatoid arthritis, etc.; ⑦ Those who have a history of shoulder surgery and a history of mental illness.

All patients participating in the study will be informed of the method, purpose and possible risks of the study. At the same time, they need to sign a written informed consent. They are free to choose whether to continue the trial at any time.

### 2.5 Study design

Eligible participants were randomly assigned to the treatment group or the control group at a ratio of 1:1 using a random tool based on the central network. Randomization was performed without any stratification generated by independent statisticians who are not involved in trial implementation or statistical analysis using SAS 9.3 software (SAS Institute, Cary, NC). The clinical research coordinator enters the participant information on the tablet and Was given a random number. The research assistant gets the participant's assignment from the computer. Throughout the research process, the research assistant is responsible for screening, recruiting participants, and assigning random numbers to the participants who have been included. The result assessor is responsible for the assessment of the scale. Except for the surgeon, all doctors, anesthesiologists, research assistants, participants, intervention supervisors, and statisticians who conduct statistical analysis, the division of personnel is unknown.

### 2.6 Intervention measures

(1) Arthroscopic evaluation: The glenohumeral joint will be examined through the posterior approach, and the rotator cuff joint will be examined through the anterior approach. The posterior approach is used to enter the subacromial space and the lateral approach was established. The shape of rotator cuff tear and the degree of tendon retraction are observed from the posterior and lateral approach respectively, and the rotator cuff tear is comprehensively evaluated. All patients are identified as full-thickness tears under arthroscopy without large and irreparable tears.
(2) Conventional arthroscopic repair: The operation will be completed by the same surgeon. Under general anesthesia, the patient takes the lateral decubitus position, disinfects, spreads the towel, and traction of affected limb. After exploring the joint cavity through the conventional arthroscopic approach, the patient enters into the subacromial region. The patients with subacromial impingement sign are given conventional subacromial decompression, and the rotator cuff break and rotator cuff insertion bone surface are freshened under the microscope, and the rotator cuff tissue is repaired with single or double row wire anchors.
(3) Implantation of the biomimetic double-layer biofilm scaffold: the bone surface of the damaged rotator cuff is freshened, and the membrane containing BMP-2 microspheres is placed on the humerus side, and the membrane containing VEGF and PDGF poly lactic-co-glycolic acid (PLGA) microspheres is placed on the tendon side.

The experimental group underwent conventional arthroscopic repair after implantation of the biomimetic double-layer biofilm stent, and the control group only underwent conventional arthroscopic repair. There were no special differences in other consumables during the operation.

### 2.7 Evaluation Criteria and Efficacy Judgment

(1) VAS:^[12]^VAS is often used to measure pain intensity.^[13]^ The patient will be asked to mark a point between 0 and 100, where 100 means maximum pain (far right) and 0 means no pain (far left);
(2) American Shoulder & Elbow Surgeons score.^[14]^ The American Shoulder & Elbow Surgeons score includes pain (50 points) and life function (50 points). The full score is 100 points. The higher the score, the better the shoulder joint function;
(3) University of California Los Angeles score:^[15]^ Including pain, function, muscle strength and range of motion of the shoulder joint. The total score is 35 points, the higher the score, the better the shoulder joint function;
(4) Constant-Murley score:^[16]^ It is the shoulder joint scoring method uniformly adopted by the European Society of Shoulder and Elbow Surgery. It consists of 4 subscales: pain (15 points), muscle strength (25 points), functional activity (20 points) and shoulder joint mobility (40 points). The total score is 100 points, the higher the score, the better the shoulder joint function.
(5) X-ray evaluation of postoperative shoulder joint anatomy, MRI evaluation of tendon anchor loosening and displacement and rotator cuff healing.
(6) Incision healing grade, incidence of adverse reactions (such as incision infection, anchor loosening, rotator cuff tearing, etc).

### 2.8 Postoperative rehabilitation and follow-up

All patients undergo rehabilitation training according to the standard plan. Postoperatively, the shoulder joint fixation device is used to fix the affected shoulder at 30° internal rotation and 20° abduction. From 0 to 6 weeks after surgery, mainly passive activities, the main actions: pendulum, forward bend, lift and external rotation, forward flexion and abduction should not exceed 90°; active exercise will be started after 6 weeks; physical labor is allowed after 6 months or sports. After 3, 6, and 12 months outpatient follow-up, X-ray and MRI examination of the shoulder joint are performed.

#### 2.8.1 Data collection and management

During each follow-up, 1 to 2 assistants will collect the follow-up data according to the evaluation criteria, and record them clearly in a pre-designed form combined with the basic information of patients. The information and data of all patients will be collected and stored in a fixed storage room to protect the confidentiality before, during and after the trial. Access to the database is limited to the researchers of this research group.

#### 2.8.2 Statistical analysis

We will use SPSS 16.0 statistical software for statistical analysis. Continuous variables were described as the mean ± standard deviation, and differences between groups were analyzed using a series of 1-way analysis of variance (ANOVA) with Bonferroni post-hoc test, while differences between groups over time were analyzed using multi-way ANOVA with Bonferroni post-hoc test. Categorical variables were described as the number (%), and were analyzed by Fisher exact test. A *P* value of < .05 was considered statistically significant.

## 3 Discussion

At present, the incidence of rotator cuff nonhealing and re-tearing after rotator cuff injury is still high.^[17]^ Most scholars believe that poor healing of the tendon at the stop of the rotator cuff is a key factor affecting the effect of surgery. In recent years, with the rapid development of tissue engineering, the repair of the tendon-bone interface of rotator cuff injuries has also made great progress. Different treatment methods have their own advantages, and the combined application of multiple strategies may be the most effective method.^[18]^

The biomimetic double-layer biofilm scaffold is prepared using natural small intestinal submucosa and chitosan, and Poly (lactic co glycolic acid) (PLGA) microspheres are used as controlled-release carriers of BMP-2, VEGF and PDGF. PLGA microspheres which could simulate the controlled release of BMP-2 in vivo and PLGA microspheres with sequential controlled release of VEGF and PDGF were combined into the inner and outer layers of the double-layer membrane scaffold respectively, and the bone marrow mesenchymal stem cells are inoculated in the periosteum The scaffold is used to construct a biomimetic periosteum with biological activity and loaded with stem cells, and then transplant it to the tendon-bone interface to promote tendon-bone healing. The biomimetic double-layer biofilm scaffold combines growth factors, repair scaffolds, and stem cells to reflect the combined application strategy. Therefore, this study will provide new ideas for the treatment of poor tendon bone healing after rotator cuff injury, and it is expected to reduce the incidence of rotator cuff nonunion and re-tear.

This study also has the following limitations: due to the influence of intervention methods, this study cannot be strictly double-blind, which may affect the results; lack of quantitative evaluation indicators for rotator cuff healing and secondary arthroscopic observation; the current protocol has a short follow-up time. If necessary, we will plan to extend the follow-up to observe the medium and long-term effects.

## Author contributions

**Data curation:** Liang Ma, Yongtao Xu, Yongtao Xu.

**Funding acquisition:** Yongtao Xu, Yongtao Xu.

**Investigation:** Xiaolong Xu.

**Resources:** Xiaolong Xu, Qin Pan.

**Software:** Qin Pan.

**Supervision:** Xiaolong Xu, Yongtao Xu.

**Writing – original draft:** Liang Ma, Yongtao Xu, Yongtao Xu.

**Writing – review & editing:** Liang Ma, Yongtao Xu, Yongtao Xu.
